# Serum 25(OH) vitamin D level and its relation to diabetic peripheral neuropathy in Egyptian patients with type 2 diabetes mellitus

**DOI:** 10.1186/s41983-018-0036-9

**Published:** 2018-11-20

**Authors:** Seham E. Abdelsadek, Entesar O. El Saghier, Sabah I. Abdel Raheem

**Affiliations:** 1 Neurology department, Faculty of Medicine for girls, AL-Azhar University, Cairo, Egypt; 2 Endocrinology and Metabolism Department, Faculty of Medicine, AL-Azhar University, Cairo, Egypt; 3 Clinical Pathology Department, Faculty of Medicine, AL-Azhar University, Cairo, Egypt; 4 Riyadh, Saudi Arabia; 5 Faculty of Medicine for Girls, AL-Azhar University, Cairo, Egypt

**Keywords:** Type 2 diabetes mellitus, Diabetic peripheral neuropathy, 25(OH) vitamin D

## Abstract

**Background:**

Some clinical studies demonstrated a significant association between vitamin D deficiency and diabetic peripheral neuropathy (DPN).

**Objective:**

This study aims to evaluate the correlation between serum levels of 25(OH) vitamin D and DPN in Egyptian patients with type 2 diabetes mellitus (T2DM).

**Patients and method:**

Sixty patients were known to have T2DM, which classified into the following: group I included 40 patients with DPN and group II included 20 diabetic patients, without DPN, compared with 30 apparently healthy subjects of matched age and sex as control group. Laboratory investigations including fasting and 2-h postprandial blood glucose levels, serum calcium, phosphorus, glycosylated hemoglobin (HbA1c), lipid profile, serum 25(OH) vitamin D, and nerve conduction studies were carried out for every participant to detect the existence and severity of DPN.

**Results:**

Vitamin D deficiency was found in 73.3% of T2DM groups and in 35% of control subjects with statistical significant differences (*p* < 0.005), and serum level of 25(OH) vitamin D in patients with DPN (21.09 ± 8.38) was less statistically significant than that in patients without DPN (31.12 ± 14.85) (*p* = 0.001). Mean serum level of 25(OH) vitamin D in patients with painless DPN (10.047 ± 8.12) was less significant than that in patients with painful DPN (18.14 ± 3.85), (*p* < 0.05). Regression analysis revealed that vitamin D deficiency is one of the independent risk factors of DPN, (OD, 0.914), (*p* = 0.007).

**Conclusion:**

Vitamin D deficiency has a significant role in the development and severity of DPN in Egyptian patients with T2DM.

## Introduction

The most common cause of peripheral neuropathy in Egypt is diabetes mellitus (DM). Diabetic peripheral neuropathy is one of microvascular complications that causes morbidity and mortality in patients with diabetes mellitus; 50% of diabetic patients have diabetic neuropathy [1]. DPN complications are foot amputation due to painless foot ulcers, and diabetic autonomic neuropathy may be associated with life-threatening conditions such as sudden death and silent myocardial ischemia [2]. Vitamin D deficiency is a common public health problem all over the world [3]. Vitamin D deficiency contributes significantly to the pathogenesis of the two types of diabetes by impairing insulin secretion from pancreatic beta-cells [4] and increasing insulin resistance [5]. Clinical studies reported that vitamin D deficiency is more common in patients with diabetes and plays an important role in pathogenesis of diabetic neuropathies [6, 7]. Clinical observational studies demonstrated a significant association among patients with vitamin D deficiency, and neuropathic pain symptoms [8], neurological deficits, autonomic dysfunction [9], and electrophysiological studies in diabetic patients [10], Also, a prospective clinical study reported the improvement of neuropathic pain in DPN patients with vitamin D supplementation [11].

### Aim of the study

To evaluate the correlation between serum levels of serum 25-hydroxyvitamin D and DPN in Egyptian patients with type 2 DM.

## Subjects and methods

### Study cohort

A case control study was carried out during the period from October 2016 to March 2017 on 80 subjects: their ages ranged from 40 to 60 years. Sixty patients with T2 DM as patients group were selected from outpatient diabetes clinic and neurology clinic of Al-Zahra University Hospital, and the control group included 20 non diabetic healthy subjects of matched age and sex. They were selected from patient’s relatives. Patients with T2DM had been classified into the following: group Ι: included 40 patients with DPN. DPN was diagnosed by clinical assessment (defined by presence at least two positive or negative sensory symptoms, signs, or reflex abnormalities) and abnormal results on nerve conduction studies (defined by the presence of at least one of amplitude, latency, F-wave, or nerve conduction velocity (NCV)) abnormalities in two or more nerves) [12]. Group II included 20 diabetic patients, without clinical evident DPN or abnormal nerve conduction studies. Inclusion criteria: all patients were known to haveT2DM, aged 40 to 60 years, duration of DM ≥ 5 years with glycated hemoglobin (HbA1c) level ≤ 10% at the screening visit. Exclusion criteria: patients with renal or liver disease, patients with infectious diseases or malignancies, subjects suffering from type 1 DM, patients with history of metabolic bone diseases, or hypothyroidism or hyperthyroidism, patients currently taking vitamin D supplementation, patients receiving medication that may alter vitamin D metabolism (antiepileptic, antituberculous, corticosteroids), patients with psychiatric disorder, and alcohol intake. Patients with peripheral neuropathy due to a non-diabetic cause and pregnant or breastfeeding female patients were excluded from the study.

### Ethical issues

All subjects were informed of the general objectives of the study, and their participation in the study was fully voluntary. Confidentiality of collected data was guaranteed to participants, and an informed consent had been obtained. The study was approved by the Ethics Committee for Clinical Research of the Faculty of Medicine for Girls, Al-Azhar University.

## Methods

All patients included in this study were subjected to full history taking, fundus examination, general and neurological assessment with special stress on the duration of diabetes, drug intake (insulin or oral hypoglycemic drugs), and the presence of subjective sensory symptoms such as pain, numbness, hypothesis, and their duration. Anthropometric measurements including weight, height, and body mass index (BMI) were calculated as weight in kilogram per height in meter, and waist circumference (WC) was measured with a tape midway between the costal margin and iliac crest in the mid-axillary line, while the subject is standing and breathing normally [13].

Assessment of peripheral neuropathy was done by neuropathy disability score (NDS) and nerve conduction studies. The NDS was established by bilateral examination of the pin–prick sensation, temperature sensation, vibration test, and Achilles tendon reflex. The sensory modalities were scored as either present = 0 or absent/reduced = 1, whereas the scoring for the ankle jerks was as follows: present = 0, present with reinforcement = 1, absent = 2, so the maximum abnormal score is 10 which indicates a complete loss of sensation to all sensory modalities and absent reflexes. A score of 0–2 implies no neuropathy, 3–5 is an indicator of mild neuropathy, 6–8 is moderate, and 9–10 is consistent with severe neuropathy [14].

Patients with DPN were classified into painful and painless DPN patients according to McGill visual analog scale (VAS).VAS is a common research tool to assess the pain, it is formed of 10 cm a straight line which provides a continuous scale for subjective magnitude estimation, and the limits of this line represent the extreme of the symptoms, so in the McGill pain index assessment 0 represents no pain and 10 the worst pain ever [15].

Nerve conduction studies (NCS) were done for all subjects by using Cadwell Sierra Wave 8155 certified to CAN\TSA EP/EMG measuring system–4 channels-version 08.11, USA. The test was explained to the patients, and their consent to perform it was obtained. Proximal and distal latencies, amplitudes, and conduction velocities of both motor and sensory nerves were recorded. Motor NCS were studied for median, peroneal, and tibial nerves, and sensory nerve action potentials (SNAP) were measured from median, and sural sensory nerves also F-waves study for median nerves were done.

Laboratory investigations were done for patients and controls including complete blood picture. Liver function, kidney function, fasting and 2 h postprandial blood glucose levels, lipid profile including serum cholesterol, triglycerides (TG), high-density lipoprotein cholesterol (HDL-C) and low-density lipoprotein cholesterol (LDL-C) were examined after fasting 14 h. Measurement of 25(OH) vitamin D serum levels were done by enzyme immunoassays using EDI. Total 25(OH) Vitamin D EIA Kit for the quantitative measurement of total serum 25-OH Vitamin D2/3 levels (Epitope Diagnostics, Inc., San Diego, CA, 92121, USA). Measurement of glycosylated hemoglobin (HbA1c) was done for patients group by quantitative calorimetric determination of glycolhemoglobin in whole blood kits supplied from Stanbio Laboratory, catalog number 0350.

### Statistical analysis

Data were analyzed using Statistical Package for Social Science (SPSS) version 20. Quantitative data were expressed using mean ± standard deviation (SD) and ranges. Qualitative data were expressed as frequency and percentage. Independent samples *t* test was used when comparing two independent groups regarding quantitative data with parametric distribution. Chi-square (*χ*^*2*^) test was used to compare between two groups with qualitative data, Pearson’s correlation coefficient (*r*) test was used to describe the degree of correlation between two variables, and the sign of correlation coefficient (+, −) defines the direction of the relationship either positive or negative. Receiver operating characteristic (ROC curve) analysis was used to find out the overall predictively of parameter in and to find out the best cut-off value with detection of sensitivity and specificity at this cut-off value. The probability and significance, *p* value < 0.05 was considered significant. *p* value < 0.001 was considered as highly significant. *p* value > 0.05 was considered insignificant.

## Results

### Demographic and laboratory characteristics of studied groups

This study has been carried out on 60 patients who were known to have T2 DM, (25 males and 35 females) with mean age (46.87.7 ± 3.54) as patient groups which sub classified into the following: group I included 40 patients with DPN and group II included 2 diabetic patients, without DPN. Twenty healthy subjects (10 males and 10 females) with mean age (mean 46.35 ± 2.25) were used as control group. Mean serum levels of FBG, PPBG, total cholesterol, LDL, and BMI were significantly higher among T2DM patients group than control group (*p* < 0.001), but serum HDL and TG had no significant difference (*p* > 0.05). T2DM patients group had lower serum level of 25(OH) vitamin D than control group with no statistical significant difference (*p* > 0.05) (Table 1).

**Table 1:** Demographic and laboratory characteristics of T2DM groups (GΙ + GII) and control group

Group variables	T2DM groups(GΙ + GII) *N* = 60	Control group *N* = 20	*t* test/*χ*^*2*^	*p* value
	Mean ± SD	Mean ± SD		
Age (years)	46.87 ± 3.54	46.35 ± 2.25	0.611	0.543
Sex				
Females	35 (58.3%)	10 (50.0%)	0.423	0.515
Males	25 (41.7%)	10 (50.0%)		
DM duration (years)	8.58 ± 5.39	–	–	–
BMI (kg/m^2^)	31.49 ± 4.01	27.17 ± 1.88	4.627	0.000
W.C (cm)	102.10 ± 12.72	83.80 ± 7.14	6.104	0.000
FBG (mg/dl)	178.32 ± 74.42	84.23 ± 9.00	5.617	0.000
2hpp (mg/dl)	235.48 ± 81.68	106.85 ± 9.62	6.998	0.000
CHO (mg/dl)	206.23 ± 58.66	165.89 ± 28.64	2.952	0.004
TG (mg/dl)	164.69 ± 87.29	130.70 ± 45.91	1.662	0.101
HDL (mg/dl)	45.19 ± 17.17	45.74 ± 11.01	− 0.133	0.895
LDL (mg/dl)	138.24 ± 46.52	105.79 ± 26.73	2.953	0.004
Ca (mg/dl)	9.43 ± 0.46	9.47 ± 0.54	0.311	0.757
Ph (mg/dl)	3.93 ± 0.36	3.69 ± 0.51	− 2.274	0.026
Creatinine (mg/dl)	0.76 ± 0.23	0.89 ± 0.21	2.116	0.038
25(OH) levels ng/ml	24.43 ± 11.84	29.83 ± 9.94	− 1.832	0.071

*DM* diabetes mellitus, *BMI* body mass index, *WC* waist circumference, *FBS* fasting blood glucose, *2hpp* 2 h postprandial blood glucose, *CHO* cholesterol, *TG* triglycerides, *HDL* high-density lipoprotein cholesterol, *LDL* low-density lipoprotein cholesterol, *Ca* calcium, *PH* phosphorus

### Cut-off level of 25(OH) vitamin D deficiencies for T2DM groups and control group

In the current study, receiver operating curve shows that cut-off level for vitamin D deficiency for T2DM groups (GΙ + GII) and control group was ≤ 28.3 ng/ml with sensitivity of 87.5%, specificity of 65%, and accuracy of 75%.

### Vitamin D status among T2DM groups and control group according to its cut-off level

This study showed that 26.7% only of T2DM groups (GΙ + GII) had sufficient vitamin D (25(OH) D > 28.3 ng/ml), in comparison to 65% of control subjects (*p* value 0.005). Vitamin D deficiency (25(OH) D ≤ 28.3 ng/ml) was detected in 73.3% of T2DM groups (GΙ + GII). While 35% only of control subjects being vitamin D deficient (*p* value 0.005).

### Demographic data, laboratory characteristics, and neurophysiological studies of patient groups

As shown in Table 2, T2DM patients with DPN (group I) were older, had longer duration of DM, higher incidence of hypertension, poorer glycemic control (higher FBG, 2hpp, and HbA1c), and more impairment of neurophysiological studies than T2DM patients without DPN (group II).

**Table 2:** Demographic data, laboratory characteristics, and neurophysiological studies of T2DM group I with DPN and group II without DPN

Group variables	T2DM group I No = 40 Mean ± SD	T2DM group II No = 20 Mean ± SD	*t* test/*χ*^*2*^	*p* value
Age (years)	47.85 ± 3.14	44.90 ± 3.54	3.287	0.002
Sex			0.857	0.355
Females	25 (62.5%)	10 (50.0%)		
Males	15 (37.5%)	10 (50.0%)		
DM duration (years)	10.87 ± 4.53	4.02 ± 3.88	5.783	0.000
FBG (mg/dl)	201.65 ± 75.18	131.65 ± 46.46	3.807	0.000
2hpp (mg/dl)	264.38 ± 81.11	177.70 ± 44.05	4.450	0.000
HbA1c %	8.31 ± 1.45	6.83 ± 1.65	3.541	0.001
Type of DPN			–	–
Painless	18(45%)	–		
Painful	22(55%)			
NDS		–		
Mild	12 (30%)			
Moderate	7 (17.5%)			
Severe	21(52%)			
Median motor nerve cv (m/s)	42.78 ± 8.72	59.05 ± 5.12	− 7.690	0.000
Median motor nerve amp (mv)	3.75 ± 1.61	6.23 ± 0.93	− 6.350	0.000
Peroneal motor nerve cv	31.85 ± 4.68	44.70 ± 4.43	− 10.200	0.000
Peroneal motor nerve amplitude	1.30 ± 0.40	3.34 ± 0.60	− 15.60	0.000
Median sensory nerve cv	31.68 ± 5.20	43.65 ± 3.99	− 9.046	0.000
Median sensory nerve amplitude (uv)	9.42 ± 4.940	24.70 ± 7.48	− 9.470	0.000
Sural sensory nerve cv	30.43 ± 6.17	42.50 ± 2.82	− 8.300	0.000
Sural sensory nerve amplitude	1.07 ± 0.42	5.43 ± 0.95	− 24.647	0.000

*NDS* neuropathy disability scale, *CV* conduction velocity

### Vitamin D status amongT2DM group I with DPN and group II without DPN

In the current study, the mean serum level of 25(OH) vitamin D in patients with DPN (group I) (21.09 ± 8.38) was highly statistically significant lower than patients without DPN (group II) (31.12 ± 14.85) with *p* value = 0.001. Also, we found that 87.6% of patients with DPN had vitamin D deficiency (25(OH)D ≤ 28.3 ng/ml) and 12.5% of them had sufficient serum level of vitamin D (25(OH)D > 28.3 ng/ml compared to patients without DPN. There were 55% with sufficient serum level of vitamin D and 45% had vitamin D deficiency (25(OH)D ≤ 28.3 ng/ml with statistical significant difference (*P* = 0.001).

### 25(OH) vitamin D levels among patient with painful PDN and patient with painless PDN

In the current study, T2DM patients with DPN divided into painful diabetic neuropathy patients (*n* = 18, 45%) and painless diabetic peripheral neuropathy patients (*n* = 22, 55%). There is statistically significant difference between patients with painless and painful diabetic neuropathy as regard vitamin D deficiency, more affected in painless neuropathy. The mean serum level of 25(OH) vitamin D in patients with painless DPN (10.047 ± 8.12) was significantly lower than patients with painful DPN (18.14 ± 3.85), (*p* < 0.05).

### Correlation of 25(OH) D level with the laboratory and neurophysiological studies in T2DM groups

This study demonstrated that the serum level of 25(OH) vitamin D has statistically significant negative correlation with severity of DPN as regard to neuropathy disability scale (NDS) and neurophysiological studies (peroneal motor nerve amplitude, median sensory nerve amplitude, sural sensory nerve amplitude, and sural sensory nerve conduction velocity) (*p* value < 0.001), also the serum level of D 25(OH) has a statistical significant positive correlation with serum calcium (Table 3).

**Table 3:** Correlation of 25(OH) D level with the laboratory and neurophysiological studies in T2DM groups

Variables	25(OH)D	
	*r*	*p* value
Age (years)	0.030	0.856
DM duration (years)	0.079	0.628
BMI (kg/m^2^)	0.113	0.487
W.C (cm)	−0.060	0.714
Ca (mg/dl)	0.286	0.027
FBG (mg/dl)	−0.103	0.528
2hpp (mg/dl)	0.009	0.956
NDS	−0.586	0.001
Median motor nerve cv (m/s)	−0.189	0.242
Median motor nerve amp (mv)	−0.001	0.994
Peroneal motor nerve cv (m/s)	−0.216	0.180
Peroneal motor nerve amp (mv)	−0.495	0.001
Median sensory nerve cv (m/s)	- 0.151	0.027
Median sensory nerve amp (uv)	−0.286	0.249
Sural sensory nerve cv (m/s)	−0.387	0.004
Sural sensory nerve amp (uv)	−0.499	0.001

### Vitamin D as independent risk factor for DPN in patients with type 2 diabetes

Regression analysis test revealed that old age, long duration of DM, and high HbA1c were the most significant risk factors for development of DPN in T2DM patients. Also, the test revealed that vitamin D deficiency is one of these independent risk factors for DPN (OD, 0.914), (*p* value = 0.007). (Table 4).

**Table 4:** Regression analysis of risk factors for DPN in patients with type 2 diabetes

Univariate analysis	*p* value	Odds ratio
HbA1c %	0.000	9.333 (2.576–33.823)
Age (years)	0.001	1.975 (1.263–3.089)
HTN (mmHg)	0.003	1.396 (1.183–1.647)
DM duration (years)	0.004	1.276 (1.080–1.507)
25(OH)D (ng/ml)	0.007	0.914 (0.856–0.976)

*HTN* hypertension

## Discussion

Egypt was listed as one of the world top 10 countries in the number of patients of diabetes. In 2013, International Diabetes Federation (IDF) estimated that 7.5 million individuals have diabetes and around 2.2 million have prediabetes in Egypt [16]. Accumulating data supposes that the prevalence of diabetes mellitus is increased with vitamin D deficiency [17]. Vitamin D deficiency increases the risk of developing T2DM and metabolic syndrome by causing pancreatic β cell dysfunction and increasing the peripheral insulin resistance [18].

The clinical studies of the effects of vitamin D deficiency on Egyptian patients with T2DM and its role in diabetic neuropathy are rare.

This study was carried out to assess the serum levels of 25(OH) vitamin D in Egyptian patients with T2DM and the correlation between serum levels 25(OH) vitamin D and DPN.

Worldwide, vitamin D deficiency affects about one billion people [19]. Although the Middle East is sunny, people living in this region (15∘ to 36∘N) have high prevalence of vitamin D deficiency [20].

Some studies in the Middle East countries indicate that 70–80% of adolescent girls in Saudi Arabia and Iran had vitamin D levels of < 25 nmol/L, while in Lebanon the figure was 32% in the same age group. Studies conducted among adults indicate a prevalence of 60–65% for vitamin D values < 25 nmol/L in Lebanon, Iran, and Jordan, and 48% for cut-off below 37.5 nmol/L in Tunisia [21].

The current study has been carried out in Cairo, Egypt located at 30° 03’ N and 31° 14′ E; we found no statistical significant difference between T2DM groups and control group as regards to absolute serum level of 25(OH) vitamin D. We measured 25(OH) D levels in the summer season, which could have been the highest level. The time of year is an important factor in measuring vitamin D levels in the diagnosis of insufficiency or deficiency.

These results are in agreement with Usluogullari and colleagues [22] and Ahmadieh and colleagues [23]. However, Sarita and colleagues [24] found that serum 25(OH) D level was significantly lower in people with T2DM when compared to healthy subjects, which may be due to regional variability and susceptibility to vitamin D deficiency.

In the current study, receiver operating curve demonstrate that cut-off level for vitamin D deficiency was ≤ 28.3 ng/ml. Vitamin D deficiency (25(OH)D ≤ 28.3 ng/ml) was found in 73.3% of T2DM groups and in 35% of control subjects with statistical significant difference.

The definition of vitamin D deficiency has been controversial, in part owing to the interpretation of surrogates associated with vitamin D status. Vitamin D deficiency has been historically defined and recently recommended by the Institute of Medicine (IOM) as a 25(OH)D below 20 ng/ml, vitamin D insufficiency as a 25(OH)D of 21–29 ng/ml, and sufficient vitamin D level as a 25(OH)D > 30 ng/ml [25].

Consistent with the present research, the FIELD study has shown that vitamin D deficiency was present in 50% of 9795 patients with type 2 diabetes, and it predicted microvascular complications [26].

Although some studies performed in sunny Middle East countries such as the study of Alhumaidi and colleagues [27], they have reported a high prevalence of vitamin D deficiency in Saudi Arabia (24° 42′ N, 46° 43′ E) among diabetics and non-diabetics individuals. Also, Haq and colleagues [28] and Al Anouti and colleagues studies done in Emirates (25° 16’ N and 55° 17′ E) [29] reported a high prevalence of vitamin D deficiency among healthy Emirati adults. The same thing has been proved in Egypt by El Badawy and colleagues’ study [30].

In the current study, the mean serum levels of 25(OH) vitamin D in patients with DPN were lower than that in patients without DPN. Also, we found that 87.6% of patients with DPN had vitamin D deficiency (25(OH)D ≤ 28.3 ng/ml) compared to patients without DPN there were 45% had Vitamin D deficiency.

Diabetic patients are at high risk of micro vascular complications including DPN, which has bad impact on the quality of life, and it is associated with high mortality [31]. In Egypt, the prevalence of neuropathy ranged from 21.9% in hospital outpatient clinics to 60% in hospital inpatients [32].

The role of vitamin D in pathophysiology of DPN, some animal studies demonstrated the associations between vitamin D deficiency and low levels of nerve growth factors (neurotrophins) which are required for the development and survival of both sympathetic and sensory neurons, and cause defective neuronal calcium homeostasis [33]. Decrease in neurotrophins and defective calcium homeostasis increase nerve damage by toxins including hyperglycemia, also vitamin D receptor modulates neuronal cells differentiation and function. So, vitamin D deficiency impairs nociceptor function, worsens nerve damage, and lowers the pain threshold [34].

Clinical studies of Alamdari and colleagues [10], Bajaj and colleagues [24], and Putz and colleagues [35], reported a significant relation between vitamin D deficiency and diabetic neuropathy. Putz and colleagues [35] recommended vitamin D supplementation in patients with diabetic neuropathy.

Celikbilek and colleagues [36] examined the relation between serum vitamin D, vitamin D-binding protein (VDBP), and vitamin D receptor (VDR) with PDN; they found patients with DN had significant lower levels of vitamin D than that in patient without DN, while there were similar values of VDBP and VDR in two groups of diabetic patients with and without DN.

In the present study, patients with DPN were divided into painful diabetic neuropathy patients (*n* = 18, 45%) and painless diabetic peripheral neuropathy patients (*n* = 22, 55%); the mean serum level of 25(OH) vitamin D in patients with painless DPN (10.047 ± 8.12) was significantly lower than that in patients with painful DPN (18.14 ± 3.85), (*p* < 0.05). Also, the serum level of 25(OH) vitamin D has statistically significant negative correlation with severity of DPN as regards to NDS and nerve conduction studies (*p* value < 0.001).

Some clinical studies demonstrated significant relation between vitamin D deficiency [7] and severity of clinical manifestation of diabetic neuropathy (sensory, neurological deficits) and also with parameters of electrophysiology studies [8].

Alamdari and colleagues’ study [10] found that the serum vitamin D level was significantly inversely correlated with the intensity of nerve conduction velocities impairment (*p* = 0.001). Also, Kheyami’s study [37] demonstrated that vitamin D receptor (VDR) expression is increased in diabetic neuropathy patients and the VDR up-regulation is associated with the severity of neuropathy and peroneal nerve conduction velocity, but there is no difference in VDR expression between painless neuropathy and painful neuropathy as demonstrated by this study. However, previous studies have suggested that vitamin D may act as an analgesic in painful diabetic neuropathy. The study done by Kheyami reported that vitamin D has analgesic effect in patients with painful DPN but vitamin D deficiency does not more associate with painful neuropathy than painless neuropathy [38].

To further examine the relationship between vitamin D and DPN, regression analysis test had been assessed. Long duration of DM, presence of hypertension, high HbA1c, and old age were the independent predictors of microvascular complications including DPN among T2DM. Besides these strong and well-known risk factors, vitamin D levels revealed a significant and independent association with DPN (OR 0.9, *p* < 0.007). Zoppini and colleagues’ study [39] found that higher serum 25(OH) D levels were independently associated with a reduced risk of prevalent microvascular complications. Also, clinical studies were done on patients with T2DM by Soderstrom and colleagues [8], Ahmadieh and colleagues [23], and Skalli and colleagues [40]. They concluded that vitamin D is an independent risk factor for development of DN. So, neuropathic pain can be relieved by replenish vitamin D deficiency, which reduces the use of medication for neuropathic pain like tricyclic antidepressant, antiepileptic, and narcotics with their often severe side effects. Also, the neuroprotective effects of vitamin D may reverse the neuronal damage and prevent the progression of DPN [6].

## Conclusion

Until now, there is only symptomatic treatment for DPN with control of blood sugar. The present study with previous clinical studies demonstrated that vitamin D deficiency has a significant role and independent association with DPN, also vitamin D serum levels correlate with the severity of neuropathy in patient with T2DM; therefore, we recommend measurement of serum levels of 25-OH vitamin D in all diabetic patients and vitamin D intake if there is vitamin D insufficiency.

## Abbreviation

BMI: Body mass index
DM: Diabetes mellitus
DPN: Diabetic peripheral neuropathy
EIA: Enzyme immunoassay
FBS: Fasting blood glucose
HbA1c: Glycosylated hemoglobin
HDL-C: High-density lipoprotein cholesterol
LDL-C: Low-density lipoprotein cholesterol
NCS: Nerve conduction studies
NCV: Nerve conduction velocity
NDS: Neuropathy disability score
PPBS: 2-h postprandial blood glucose
SNAP: Sensory nerve action potentials
TG: Triglycerides
VAS: McGill visual analog scale
VDBP: Vitamin D-binding protein
VDR: Vitamin D receptor
WC: Waist circumference
